# Prise en charge d’un lipome cervical géant: à propos d’un cas et revue de la littérature

**DOI:** 10.11604/pamj.2021.39.100.12727

**Published:** 2021-06-03

**Authors:** Mohammed Elakhiri, Youssef Darouassi, Mohammed Oukabli, Ali Jahidi, Fouad Benariba

**Affiliations:** 1 Service d’Oto-Rhino-Laryngologie et Chirurgie Cervico-Faciale, Hôpital Militaire Avicenne, Marrakech, Maroc,; 2 Service d’Anatomopathologie Hôpital Militaire d´Instruction Mohammed V, Rabat, Maroc,; 3 Service d’Oto-Rhino-Laryngologie et Chirurgie Cervico-Faciale, Hôpital Militaire d´Instruction Mohammed V, Rabat, Maroc

**Keywords:** Lipome cervical géant, tumeur, rapport de cas, Giant cervical lipoma, tumor, case report

## Abstract

Le lipome est la plus fréquente des tumeurs des parties molles, elle survient rarement au niveau de la tête et du cou. Une lésion de grande taille (> 10cm) avec un taux de croissance rapide devrait soulever des inquiétudes sur une malignité possible. Nous rapportant le cas d´un patient qui s´est présenté avec un lipome cervicale inhabituel par sa taille d´environ 46cm, diagnostiqué en imagerie notamment la tomodensitométrie (TDM). La prise en charge thérapeutique est chirurgicale.

## Abstract (en)

Lipoma is the most common of soft tissue tumours. It rarely occurs in the head and neck. Patients with fast-growing large sized lesion (> 10cm) should be suspected to have a cancer. We here report the case of a patient presenting with unusual cervical lipoma (size: approximately 46cm), diagnosed based on imaging tests, including computed tomography (CT) scan. Patient´s management was based on surgery.

## Introduction

Le lipome est la plus fréquente des tumeurs des parties molles, elle survient habituellement dans les régions riches en tissus adipeux (tronc, épaule, membre supérieur), plus rarement au niveau de la tête et du cou. Les lipomes géants sont définis par Sanchez *et al*. comme des lésions dont la taille est inférieure à 10cm et dont le poids est inférieur à 1000g [1]. Une masse cervicale de grande taille (> 10cm) avec un taux de croissance rapide devrait soulever des inquiétudes sur une malignité possible [1]. Le diagnostic positif repose essentiellement sur la TDM (tomodensitométrie) et l´IRM (imagerie par résonnance magnétique), Le traitement est essentiellement chirurgical. Nous rapportant le cas d´un patient qui s´est présenté avec une masse cervicale inhabituelle par sa taille d´environ 46cm dont le bilan initial était en faveur d´un lipome; le patient a bénéficié d´un traitement chirurgical avec un bon résultat esthétique sans déficience fonctionnelle.

## Patient et observation

**Figure 1:**
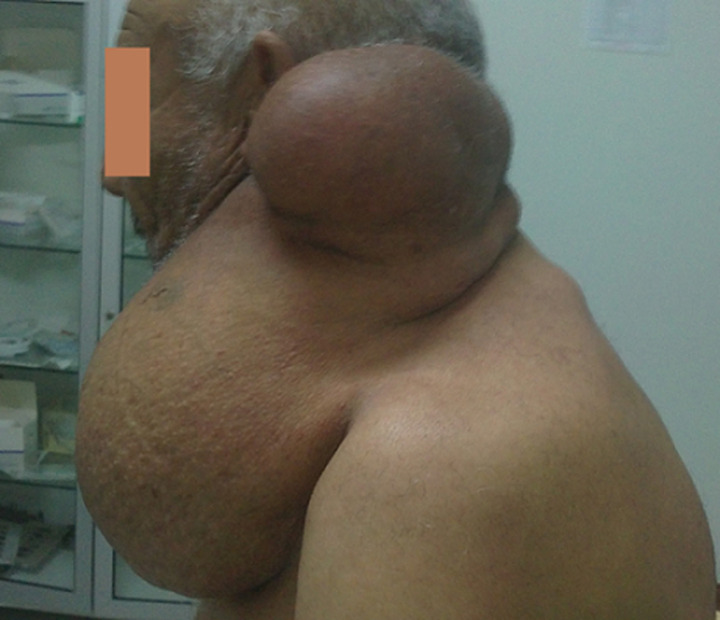
lipome cervical géant (vue de profil)

Il s´agit d´un patient âgé de 70 ans, sans antécédents pathologiques qui s´est présenté avec une masse latérocervicale gauche évoluant depuis plus de 20 ans augmentant progressivement de taille sans syndrome compressif. L´examen clinique a objectivé à l´inspection une énorme masse allant de la mastoïde au 1/3 supérieure de l´hémi-thorax gauche, ovoïde régulière à grand axe vertical d´environ 45cm avec une ectasie veineuse de surface et sans ulcération de surface. La palpation a retrouvé une masse bilobée avec une large base d´implantation, de consistance ferme et indolore (Figure 1). L’échographie cervicale a trouvé l´aspect évocateur de lipome avec un caractère régulier homogène et quelque zone de nécrose, le tout enveloppé par une capsule bien limitée, cette masse refoule et comprime les vaisseaux du cou sans les infiltrer. La TDM a confirmé les données de l´échographie en montrant une densité graisseuse homogène encapsulée sans communication avec la moelle épinière. L´IRM n´a pas pu être réalisé vue la taille de la masse cervicale. L´étude cytologique après cytoponction à l'aiguille fine a révélé la présence de lipocytes matures indicatives de lésion lipomateuse.

**Figure 2:**
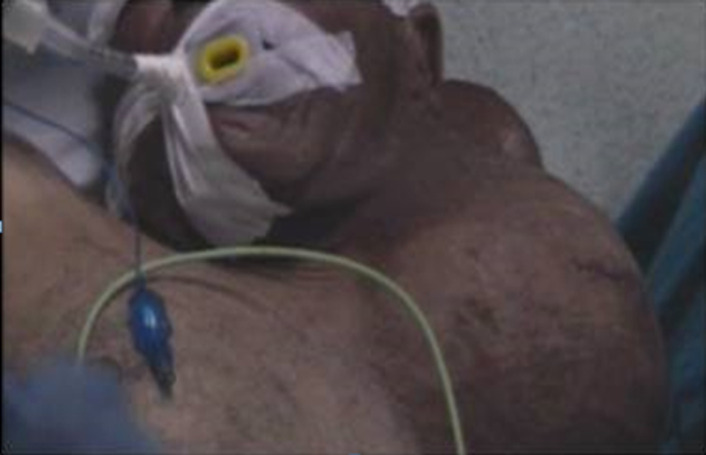
patient en décubitus dorsale (anesthésie générale)

**Figure 3:**
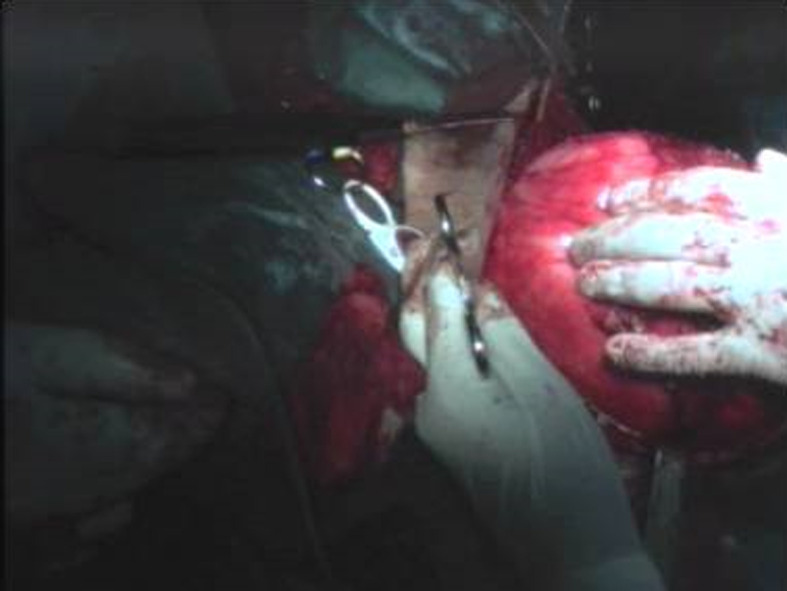
vue peropératoire

**Figure 4:**
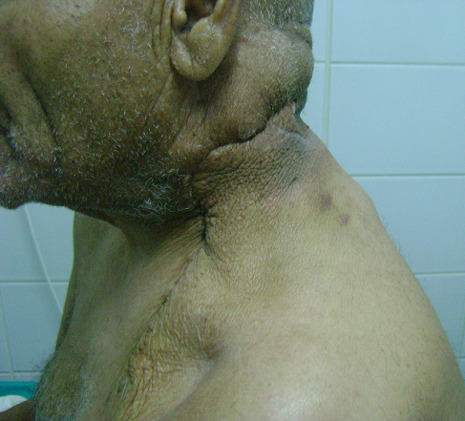
aspect postopératoire

**Figure 5:**
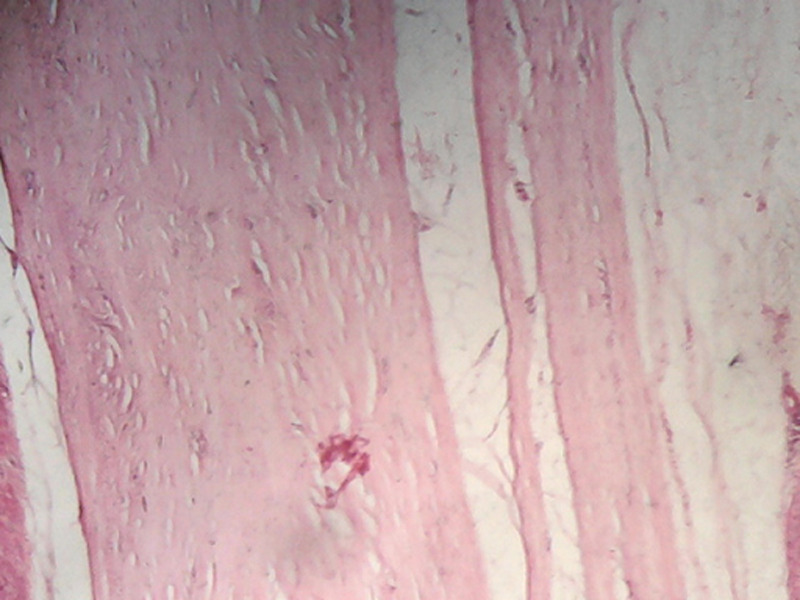
prolifération faite d´adipocytes matures sans atypies cytonucléaires et sans lipoblaste (HEx 100)

**Figure 6:**
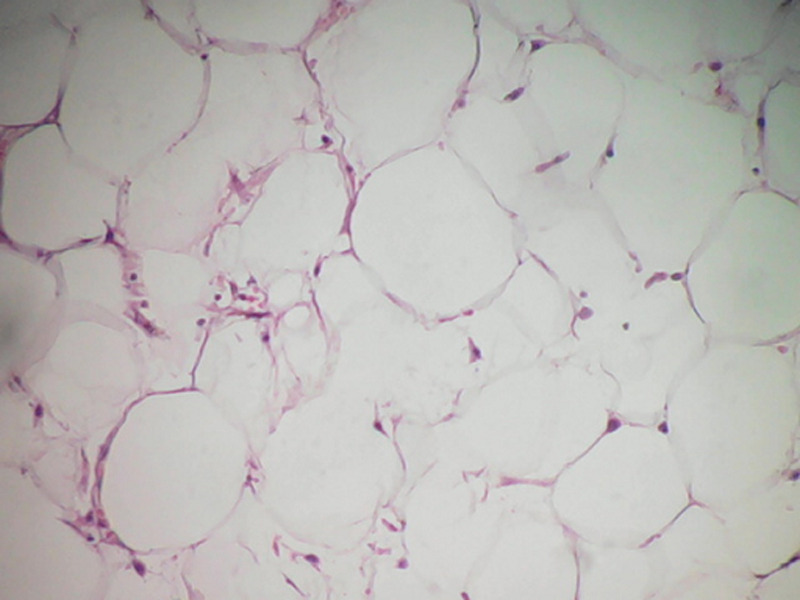
la prolifération est entourée par une capsule fibreuse épaisse sans infiltration tumorale (HEx 10)

**Figure 7:**
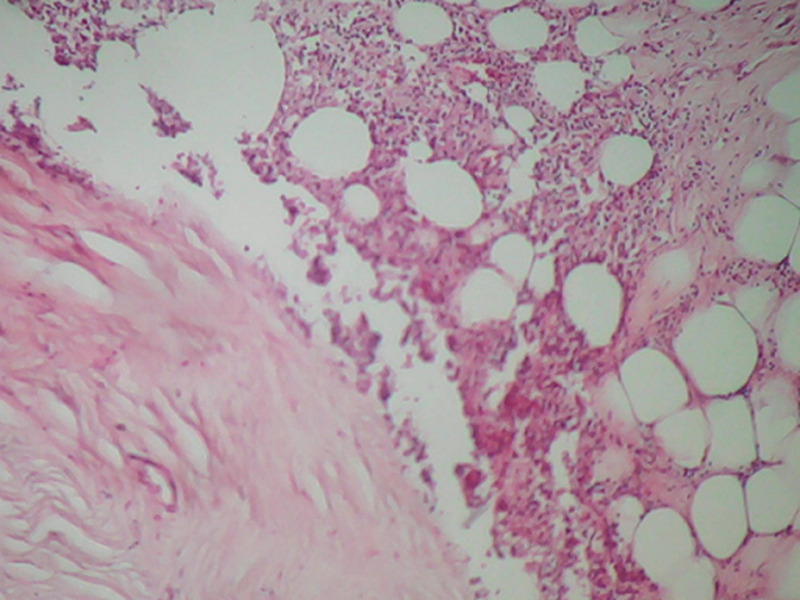
remaniements fibreux hyalins acellulaires parfois associés à des foyers de stéatonécrose au sein de la tumeur (HEx 10)

Le patient a subi une exérèse chirurgicale de la masse cervicale sous anesthésie générale (Figure 2). La dissection de la masse a été réalisée selon un plan de clivage (Figure 3). Le résultat esthétique a été jugé satisfaisant et aucune déficience fonctionnelle n´a été notée en postopératoire (Figure 4). L´étude histologique de la pièce opératoire a montré une prolifération tumorale - limité par une capsule fibreuse épaisse (Figure 5) - faite d´adipocytes matures sans atypies cytonucléaires et sans lipoblaste visible (Figure 6) et comportant par endroits des remaniements fibreux hyalins acellulaires parfois associés à des foyers de stéatonécrose (Figure 7). A noter l´absence totale des signes en faveur de la malignité. Les suites postopératoires ont étés simples.

## Discussion

Le lipome est une tumeur bénigne formée de globules graisseux. C´est la plus fréquente des tumeurs mésenchymateuses. Elle se présente comme une tuméfaction souple ou molle située sous la peau, insensible mais parfois gênante en raison de sa situation ou de son volume, typiquement asymptomatique et de croissance lente. Elle est le plus souvent superficielle et solitaire mesurant dans 80% des cas moins de 5cm (moins de 1% mesurent plus de 10cm) [2]. Les lipomes profonds sont moins fréquents et paraissent moins bien délimités dans les localisations intra et intermusculaire ou rétropéritonéale. Les localisations les plus fréquentes, en ordre décroissant de fréquence, sont le dos, les bras, les épaules, la paroi thoracique antérieure, le sein, la cuisse, la paroi abdominale, les jambes, le front et le visage [3]. Seuls environ 25% surgissent dans la tête et du cou [4] surtout au niveau de la région postérieure. Ces tumeurs sont plus fréquentes chez les femmes et surviennent habituellement au cours de la quatrième et cinquième décennie.

La plupart des lipomes ne posent aucune difficulté diagnostique. Toutefois, devant de grandes masses (> 10cm) ou une croissance rapide, et surtout au niveau de la région tête et cou, on doit penser à une tumeur maligne. Rarement, les lipomes peuvent être initialement malin ou le devenir avec l´évolution [5]. Des complications sont également possibles à type de saignement d'ulcère surtout avec les lipomes géants dans la région du cou. Le diagnostic a bénéficié de l´amélioration des techniques. A l´échographie, la masse est homogène dans 2/3 des cas, avec a un grand axe parallèle à la peau et un rapport grand axe/petit axe supérieur à 3. A la TDM, La densité est de type graisseux avec ou sans capsule fibreuse. L´IRM objective une masse de même signale que la graisse sous-cutanée avec un hypersignal T1 spontané et peut contenir de fine septa (<2mm). Les lipomes intramusculaires, ne possédant pas de capsule, se présentent souvent avec des limites irrégulières et des interdigitations faites de fibres musculaires, responsables d´un aspect strié. Les lipomes intermusculaires peuvent engainer les axes vasculo-nerveux (comme par exemple le creux poplité). Rarement, la présence de plages d´infarctus ou de nécrose au sein du lipome peuvent faire suspecter un liposarcome. Par ailleurs, les limites du lipome sont clairement définies par l'IRM permettant de le distinguer du tissu adipeux environnant, une distinction qui ne peut être obtenue à la TDM.

Le traitement de choix est l'exérèse complète qui ne pose généralement pas de difficultés en raison de la présence d´un pseudo capsule bien définie. La confirmation du diagnostic se fait par l´étude anatomopathologique. Macroscopiquement, les lipomes se présentent sous forme d´une masse molle, jaunâtre, brillante, lisse, mobile, encapsulé et avec éventuellement de fines cloisons. Au microscope, les lésions montrent une croissance lobulaire des adipocytes matures avec des frontières délimitées, une capsule fibreuse et une vacuole centrale [6]. L´évolution généralement est bonne, les récidives surviennent dans 4 à 5% des cas surtout pour les lipomes infiltrants ou profonds.

## Conclusion

Le lipome est une tumeur bénigne facile à diagnostiquer en imagerie notamment la TDM et d´IRM. La prise en charge thérapeutique est chirurgicale et ne pose habituellement pas de problèmes particuliers. C´est une tumeur peu évolutive mais qui peut discrètement augmenter de volumes au fil des années ou des décennies.
